# 09 Validation of the francophone version of pGALS with the Delphi approach

**DOI:** 10.1093/rheumatology/keac496.005

**Published:** 2022-10-07

**Authors:** Ferjani Hanen, Rabhi Emna, Loucif Lilia, Hadef Djohra, Tekaya Rawdha, Slimani Sami, Hamdi Wafa, Helen Foster

**Affiliations:** 1 Kassab Orthopedics Institute, Rheumatology Department, Ksar Said, Tunisia; 2 Department of Paediatrics, Batna 2 University, Batna, Algeria; 3 Charles Nicolle Hospital, Department of Rheumatology, Tunis, Tunisia; 4 Atlas Clinic of Rheumatology, Batna, Algeria; 5 Population and Health Sciences Institute, Newcastle University

## Abstract

**Background:**

The pediatric Gait, Arms, Legs, and Spine (pGALS) is a practical quick musculoskeletal assessment to increase awareness of the joint disease, facilitate early recognition of joint problems, and prompt referral to specialist teams to optimize clinical outcomes. pGALS has been shown to be practical and useful, with excellent acceptability by children and their parents. Its use was limited in French-speaking countries because of the lack of the francophone version.

**Objectives:**

To describe the steps of the translation of the pGALS francophone version using the Delphi approach.

**Methods:**

Delphi method is the consensus-building method, providing the consensual opinion of the experts. For each translated item of the pGALS, the experts assessed the relevance using a scale ranging from 1 to 9 (not relevant-completely relevant). Then median was calculated giving for each item the position of the group: disagree (if the median < 3), equivocal (median between 4–6) and agreement (median >7). The degree of the convergence with the group was assessed to clarify this result: the group’s opinion is consensual if 70% of the responses were within the range of the median; otherwise, it’s “not consensual”. For the no consensual and no relevant item, the experts propose a comment to reformulate the sentence.

**Results:**

Three native speakers were invited to translate the English form of the pGALS into the francophone language. The different propositions were mixed in a consensual way by a children’s musculoskeletal specialist. The version was validated according to the Delphi method. Six experts (pediatricians and rheumatologists) from different French-speaking countries were interviewed during 3 rounds by electronic survey individually and anonymously. After each round: the median, consensus, and comments of every item are collected and a meeting with experts was held to analyze the results. During the first meeting, we were consensual and we had an agreement on 82% of the items (28 items were validated, and 6 items were reformulated). Then the form was reformulated using the results of the preliminary rounds: opinions of the experts and their proposals during the last meeting). We were in agreement and we validated the remaining six-item during the second meeting. In the last round, we obtain a consensual version of pGALS.

**Conclusion:**

Our approach contributed to the consensual translation of the francophone version of pGALS. This tool is now ready to be used as a basic clinical skill. More research is mandatory to assess its sensibility and specificity in screening musculoskeletal disorders.

